# A decade of antimicrobial resistance in *Vibrio* spp.: genomic and functional insights

**DOI:** 10.1128/spectrum.02162-25

**Published:** 2026-04-02

**Authors:** Agila Kumari Pragasam, Chandana Basak, Mansi Rajwanshi, Lekshmi Narendrakumar, Goutam Chowdhury, Prabhakar Babele, Meenal Chawla, Orlando Moranchel, Upma Singh, Deboleena Roy, Pradipta Jana, Tanshi Mehrotra, Debaleena Das, Daizee Talukdar, Deepjyoti Paul, Rupak K. Bhadra, Thandavarayan Ramamurthy, Amit Ghosh, Shanta Dutta, Christophe Possoz, François-Xavier Barre, Asish K. Mukhopadhyay, Bhabatosh Das

**Affiliations:** 1 Centre for Microbial Research, BRIC-Translational Health Science and Technology Institute, NCR Biotech Science Cluster 145787, Faridabad, India; 2 Bacterial division, ICMR-National Institute for Research in Bacterial Infections 30170 , Kolkata, West Bengal, India; 3 Evolution and Maintenance of Circular Chromosomes, Institute of Integrative Biology of the Cell 531573, Gif-sur-Yvette, France; 4 Infectious Diseases and Immunology Division, CSIR-Indian Institute of Chemical Biology 30156, Kolkata, West Bengal, India; Jawaharlal Nehru Centre for Advanced Scientific Research, Bangalore, Karnataka, India

**Keywords:** antimicrobial resistance, phylogenetic analysis, *Vibrio cholerae*, *Vibrio paracholerae*, comparative proteomics

## Abstract

Environmental pressures and repeated exposure to antimicrobials drive microbial evolution, often through genomic changes or acquisition of mobile genetic elements that enhance fitness and resistance. This has critically influenced the control of cholera, particularly with the emergence of multidrug-resistant *Vibrio cholerae* strains, such as those recently reported in Yemen. To explore the long-term dynamics of antimicrobial resistance (AMR), we analyzed 149 clinical *Vibrio* isolates collected in India over 16 years (2008–2023), including five cases of *Vibrio paracholerae*. Phenotypic testing revealed widespread multidrug resistance, while whole-genome sequencing (WGS) uncovered both known and atypical AMR determinants. Phylogenomic analysis showed diverse clustering patterns, with pan-genome comparisons identifying species-specific genomic regions despite overall high similarity. Notably, *V. paracholerae* lacked the canonical cholera toxin gene (*ctxAB*) but harbored alternative virulence factors, including *hlyA*, *chxA*, proteases, and type VI secretion system components, supporting its role as a non-cholera enteric pathogen of concern. Our study underscores the importance of long-term surveillance and comparative genomic profiling to understand AMR evolution and guide cholera management in endemic regions.

**IMPORTANCE:**

This study highlights how long-term environmental and antimicrobial pressures shape the evolution of *Vibrio* species, leading to multidrug resistance and genomic diversification. By analyzing 16 years of clinical isolates from a major cholera-endemic region in India, including emerging *Vibrio paracholerae* cases, we reveal both known and novel resistance mechanisms and virulence factors. These findings emphasize the need for sustained genomic surveillance to track evolving resistance trajectories and support effective control strategies for cholera and related enteric infections in endemic regions.

## INTRODUCTION

*Vibrio cholerae* is the causative agent of the diarrheal disease called cholera, which remains one of the major public health concerns in the developing countries. Recurrent large-scale outbreaks pose a threat to public health, particularly in regions with limited access to clean water and sanitation (1). Cholera typically causes acute watery diarrhea and can be fatal without treatment. While treatment with oral rehydration solution and antibiotics in severe cases can reduce illness duration, the rise of antimicrobial resistance (AMR) has limited treatment options (2). Over the years, the understanding of *V. cholerae* infection and its evolution in response to antimicrobial agents has become a critical area of research.

*V. cholerae* strains can be classified into >200 serogroups based on the lipopolysaccharide O-antigen, highlighting their genomic plasticity. The O1 and O139 serogroups are the most prevalent in epidemic and pandemic regions. The current ongoing pandemic (1961 to date) is due to O1 El Tor strains, referred to as seventh pandemic El Tor (7PET) (3, 4). The clinical significance of *V. cholerae* is due to the production of cholera toxin (ctx) and the presence of several acquired AMR genes, emphasizing the role of mobile genetic elements (MGEs) in its pathogenic evolution (5, 6). The genomic plasticity of *V. cholerae* facilitated the acquisition of MGEs through gene exchange events, leading to the pathogen’s evolution and its persistence. This includes the regions encoding for *Vibrio* seventh pandemic (VSP-1 and VSP-2) and distinct bacteriophage CTXΦ in 7PET, besides the presence of *Vibrio* pathogenicity islands (VPI-1 and VPI-2) of classical O1 strains (7, 8). Within the 7PET lineage, the phylogenetic analysis identified three sub-clades with independent transmission waves referred to 7PET as waves 1–3 (9). Within these sub-clades, strains of wave 2 and 3 harbored a self-transmissible integrative conjugative element (ICE) that carried multiple AMR genes. Similarly, O139, which emerged during the 1990s, also contained ICE (referred to as SXT-ICE), encoding resistance genes like *dfrA*, *sul2*, *strAB*, and *floR* (10, 11). Serogroups other than O1 and O139 are collectively called non-O1/O139 and mostly they lack genes encoding for virulence, causing sporadic mild infections.

*Vibrio paracholerae*, classified as a novel species in 2021, is eventually raising concern due to its discovery from water systems, animal feed, and clinical samples like stool and wound (12). *V. paracholerae* is different from *V. cholerae* in terms of lacking the major virulence factors like CTXΦ, VPI-1, VPI-2, and ctx making it incompatible to cause cholera. However, the presence of genes encoding virulence factors like RTX toxin, cholix toxin, cytolysin HlyA, OmpU, secretory systems, and different proteases in some of the *V. paracholerae* strains warrants further investigation (13). Although this species is not well documented as a waterborne human pathogen like cholera, its presence in aquatic bodies and isolation from human stool samples suggests that this bacterium has a potential adaptation strategy to colonize in human gut.

The rising antibiotic resistance in epidemic and pandemic *V. cholerae* strains has severely limited treatment options and hindered control efforts, as seen in the recent emergence and spread of a multidrug resistance plasmid in the seventh pandemic El Tor *V. cholerae* isolates from Yemen (14) and Mayotte (15). Additionally, the emergence of *V. paracholerae* raises further concerns about its potential pathogenicity. To address this, we conducted an integrative genomic and proteomic analysis of clinical *V. cholerae* and *V. paracholerae* isolates collected over 16 years (2008–2023) from a cholera-endemic region, aiming to assess the impact of gene flux on pathogen evolution, providing critical insights for future surveillance and disease management strategies.

## MATERIALS AND METHODS

### Bacterial isolation and antimicrobial susceptibility testing

Among the clinical isolates of *Vibrio* spp. collected from stool samples of diarrheal patients at National Institute for Research in Bacterial Infections between 2008 and 2023, a total of 154 representative isolates were included in the study, with 8–10 isolates each year. All the isolates were phenotypically confirmed as *Vibrio* spp. as per the standard biochemical testing methods and antisera agglutination methods using *V. cholerae* O1 polyvalent Ogawa, and Inaba monovalent antisera (Denka-Seiken, Tokyo, Japan) for serogroup identification (16). These were further sent to the Functional Genomics Laboratory at the BRIC-Translational Health Science and Technology Institute (THSTI), Faridabad, where phenotypic antimicrobial susceptibility testing and genome sequencing studies were carried out.

Phenotypic susceptibility testing was done by Kirby-Bauer disk diffusion as per Clinical and Laboratory Standards Institute (CLSI) recommended testing method for the following antibiotics (Liofilchem): ampicillin (AMP) 10 μg , chloramphenicol (CHL) 30 μg, trimethoprim (TMP) 5 μg, sulfamethoxazole (SFX) 50 μg, nalidixic acid (NA) 30 μg, ciprofloxacin (CIP) 5 μg, spectinomycin (SCM) 100 μg, streptomycin (SPM) 10 μg, tetracycline (TET) 10 μg, gentamicin (GEN) 10 μg, kanamycin (KAN) 30 μg, neomycin (NEO) 10 μg, erythromycin (ERY) 15 μg, rifampicin (RFM) 30 μg, aztreonam (AZT) 30 μg, and imipenem (IMP) 10 μg. The diameter of the zone of inhibition was interpreted as per CLSI guidelines interpretative criteria, 2021 (17). 

### Whole-genome sequencing, data processing, and quality check

Genomic DNA extraction was performed by growing the isolates overnight in Luria-Bertani (LB) broth followed by using the genomic DNA extraction kit according to the manufacturer’s instructions (GenElute Bacterial Genomic DNA Kits, Sigma-Aldrich). The whole-genome sequencing of the culture-purified samples involved library preparation steps using the Illumina Nextera XT library preparation kit (#FC-131-1096). Samples requiring a minimum quantity of 1 ng, as measured with the Qubit Flex Fluorometer, underwent tagmentation through *in vitro* transposition (18), breaking down into smaller fragments measuring between 300 and 1,000 base pairs. DNA fragments were indexed for library preparation using the Illumina Nextera XT V2 index kit (#FC-131-2001,02,03,04). The library was subsequently purified with Ampure XP beads (19) and assessed for quality using the TapeStation 4200 automated electrophoresis analyzer. Any library fragment populations smaller than 200 bp were further cleaned up with the SPRIselect Bead-Based Reagent (Beckman Coulter# B23318). All cleaned-up libraries after quality analysis were normalized to a concentration of 4 nM and pooled for sequencing at 750 pM on the Illumina Nextseq 2000 sequencing platform. A 300-cycle chemistry (R1 150 and R2 150 cycles) in the P2 300 flow cell was in the sequencing platform for the high throughput sequencing.

### Screening of AMR determinants, MGEs, and multi-locus sequence typing profiling

Sequence type of all study isolates was assigned using the PubMLST scheme using the seven housekeeping genes; *adk*, *gyrB*, *mdh*, *metE*, *pntA*, *purM*, and *pyrC* (20). Furthermore, the genome assemblies were screened for the presence of acquired antimicrobial resistance determinants through the abricate tool (https://github.com/tseemann/abricate). Genome-genome comparisons were made using the BLAST-ATLAS tool (https://paulstothard.github.io/cgview). Genomic arrangements of the region of interest were studied using the web-based server Proksee (https://proksee.ca/projects/). For selected bacterial isolates, the presence of virulence genes was assessed through the abricate tool using virulence factor database (https://github.com/tseemann/abricate). Also, nucleotide BLAST was performed to verify the presence of particular virulence genes.

### Genotype-phenotype concordance analysis of AMR determinants

All 154 isolates were analyzed to assess concordance between genotypic AMR determinants and phenotypic susceptibility results, treating phenotype (R/S) as the gold standard. Resistance genes were converted to binary presence/absence indicators, and where multiple genes could confer resistance to the same antimicrobial class, combined markers were created (e.g., *catB9* or *floR* for chloramphenicol; *dfrA*1 or *dfrA15* for trimethoprim; *gyrA* or *parC* mutations for fluoroquinolones; *strA* or *strB* for streptomycin). Concordance was evaluated by calculating true positives (TPs), false positives (FPs), false negatives (FNs), and true negatives (TNs). TP represented isolates in which both the gene and resistant phenotype were present; FP indicated gene positive but phenotypically susceptible isolates; FN reflected resistance in the absence of the gene; and TN indicated isolates lacking both the gene and the resistant phenotype.

### Global phylogeographical analysis

To obtain an initial overview of the species-level relationships between *V. cholerae* and *V. paracholerae*, an rRNA-based maximum-likelihood tree was generated. The 16S, 23S, and 5S rRNA sequences of the *V. cholerae* reference genome N16961 were used as reference, against which the study isolates were mapped, and the sequences were retrieved, followed by RAxML tree generation using the GTR-GAMMA nucleotide substitution model with 100 bootstrap replicates (21). For a detailed sub-lineage resolution and a global comparison, a whole-genome-based approach was considered, for which core-genome single nucleotide polymorphism (SNP)-based maximum-likelihood tree was generated. To assemble the global data set, *V. cholerae* genomes submitted at NCBI pathogen detection have been downloaded and assessed for quality check by checkM tool. Genomes that had >90% completeness and <5% contamination were included. The assemblies were subjected to screening of species and serogroups as follows. *V. cholerae* was identified through the presence of species-specific marker gene *ompW* using CP000626 (404919–405506) as a reference. The serogroup of the genomes included in the analysis was predicted *in silico* based on screening of the presence of *rfbV* and *wbfR* genes that are specific for O1 and O139 serogroups, respectively (22). The serogroup-specific gene sequences were taken from AE003852.1 (264254–265486) and AB012956 (32623–34533) and used as a reference and screened against all the study isolates using Snippy tool version 4.6.0. Following this, the number of genomes was reduced by using a dereplicator tool with a cut-off of 0.01 (https://github.com/rrwick/Assembly-Dereplicator), to choose representative genomes from the global collection. Initially, Prokka was used for annotation, and the .gff files underwent pan-genome analysis with panaroo, producing a core gene alignment from genes present in 98% of the analyzed genomes (23). This alignment served as the basis for generating a SNP-based ML tree using RAxML with standard parameters based on GTR-GAMMA and 100 bootstrap replicates (21). The resulting tree was visualized and annotated in iTOL. The pan-genome analysis obtained by Panaroo was visualized in Phandango and analyzed using the Twilight program (24).

### Sample preparation for proteomic assay

Thirteen *Vibrio* isolates were selected for proteomic analysis, including eight *V. cholerae* and five *V. paracholerae* isolates. The *V. cholerae* isolates belong to the following serogroups: O1 El Tor (MV35962, MV14585, IDH10941, and N16961), O1 classical (VCO39), non-O1/O139 (IDH9406 and BCH13807), and O139 (BCH11679). All 13 isolates were cultured in triplicate using LB broth and Alkaline Peptone Infusion (AKI) medium following the AKI-SW method (25). In AKI medium, isolates were grown under static condition for 4 hours, then in shaking condition for 16 hours at pH 7.2–7.4 and temperature 37°C. Similarly, for LB medium, isolates were incubated overnight in shaking condition at pH 6.5 (buffered with Tris) at 30°C. After incubation, samples were pelleted down by centrifuging at 4°C, dried, and stored at –80°C for further processing.

### In-solution digestion followed by protein sequence analysis using liquid chromatography with tandem mass spectrometry

In-solution tryptic digestion of the protein samples was carried out as per the method described by Babele et al. (26). Briefly, 50 µg protein was denatured by 8 M urea, and the reaction mixture was made up to 50 µL with 50 mM ammonium bicarbonate (ABC). A total of 10 mM dithiothreitol was used for reduction for up to 1 h at 37°C, followed by alkylation with 20 mM iodoacetamide in the dark. A total of 50 mM ABC was added to the sample mixture to dilute the urea concentration to 2 M. Proteins were digested with trypsin at 1:50 overnight at 37°C. Following digestion, the resulting peptide mixture was then acidified using formic acid, desalted, and vacuum concentrated.

Liquid chromatography with tandem mass spectrometry was done using a Vanquish Neo UHPLC System, connected on-line with an Orbitrap Exploris 480 Mass Spectrometer (Thermo Scientific). The trypsin-digested samples were loaded on a Trap (PepMap Neo Trap Cartridge 100 A, 5 μm C18 0.3 mm × 5 mm) and analytical nano column (Easy-Spray PepMap Neo 2 μm C18 75 μm × 150 mm). The mobile phase for HPLC was as follows: water/acetonitrile/formic acid (A, 98/2/0.1%; B, 2/98/0.1%). Sample (1 µg) was injected on column with a flow rate of 300 nL/min. Analytical separation was established by the following gradient condition: initial 8% B for 2 min, followed by a linear gradient from 8% B to 30% B in 88 min, followed by another linear gradient to 40% B in 10 min. Following the peptide elution window, the gradient was increased to 80% B in 1 min and held for 4 min. Initial chromatographic conditions were restored in 1 min and maintained for 4 min.

A data-dependent acquisition experiment was performed on an Orbitrap Exploris 480 system having a FAIMS Pro Interface. The sample data were acquired using a nano-spray ionization ion source with a voltage 1,900 V, ion transfer tube temperature 280°C, FAIMS voltages −50°C and −70°C, RF lens 40%, automatic gain control 300%, maximum injection time in auto mode, and one microscan. The MS was operated at a resolution of 60,000 FWHM (200 m/z) and a mass range of m/z 350–1,200. A total of 30 survey scans were acquired at 28% HCD collision energy if exceeding an intensity threshold of 5.0e^3^ with a 2+ to 6+ charge states. Dynamic exclusion duration was set for 45 s.

### Bioinformatics analysis

Identification of proteins in the samples was performed via database searching against the UniProt *V. cholerae* and *V. paracholerae* protein database (August 2024) with Proteome Discoverer software having SequestHT algorithm. The search parameters were as follows: enzyme trypsin; maximum missed cleavage 2; precursor mass tolerance 10 ppm; fragment mass tolerance 0.02 Da; static modification Cys-carbamidomethyl; dynamic modification Met oxidation; target/decoy strategy using percolator; target false discovery rate 1%. Protein intensities were calculated at precursor level utilizing already provided nodes in the Proteome Discoverer software using SequestHT with Percolator validation.

### Bioinformatics analysis of *dif* sequences

The presence of *dif1* sequences and *difG* sequence was analyzed among the strains of *V. cholerae* and *V. paracholerae* using NCBI BLASTn (27). Hits were considered positive only if the aligned region showed 100% nucleotide identity over the full length. The *dif1* sequences of the following strains N16961, O395, H25, H12, WO5, Ku-40, 93,333, AM19226, 1587, and RC385 were considered for this study. The *difG* sequence of non-toxigenic *V. cholerae* O1 and non-O1/O139 was evaluated for this study (28).

## RESULTS

### Genome-based species prediction revealed the presence of atypical *Vibrio* spp. in clinical samples from patients with diarrhea

**Fig 1:**
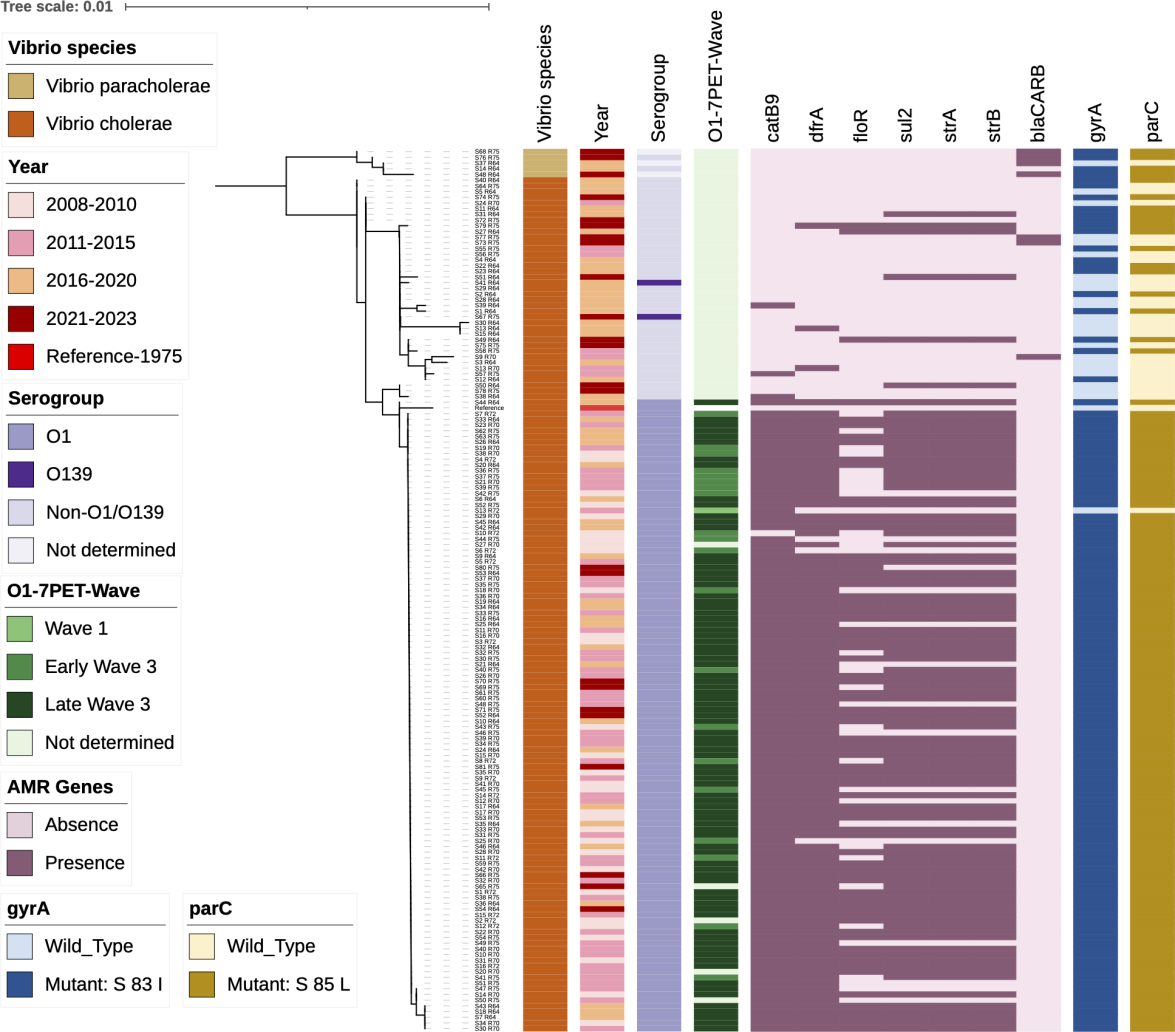
Maximum likelihood tree based on 16S, 23S, and 5S rRNA gene sequences of the study isolates (*n* = 154) mapped against the reference sequence of *V. cholerae* N16961. Color bar refers to the *Vibrio* spp., year of isolation, serogroup, and 7PET-Waves. A heatmap represents the presence (dark shade) and absence (pale shade) of the acquired AMR determinants; the chromosomally encoded genes *gyrA* and *parC* with wild type and mutated bases. Tree scale indicates the number of substitutions per genome per site (see also Table S1).

This study included 154 isolates from clinical stool samples of diarrheal patients, which were further identified as *V. cholerae* (*n* = 149), and *V. paracholerae* (*n* = 5). *In silico* genome-based serogroup prediction of *V. cholerae* isolates revealed the predominance of O1 (*n* = 110), followed by non-O1/O139 (*n* = 37) and O139 (*n* = 2) serogroup. Most O1 isolates carried the *ctxB*-7 variant, linked to late wave 3 of the current pandemic, while the remaining majorly had *ctxB*-1, associated with early wave 3. While early wave 3 strains circulated from 2008 to 2015, late wave 3 strains showed a temporal overlap signal spanning the period from 2008 to 2023. Interestingly, one isolate from 2012 has been genotyped as 7PET wave-1 (MV30946), which was thought to be extinct in the epidemiological landscape after the 1990s. Surprisingly, a few O1 isolates lacked the *ctxB* gene, which made it difficult to assign them to a specific wave. Evolutionary relationship showed that these *ctxB*-negative isolates did not form any distinct cluster; rather, they were intermixed within *V. cholerae* O1 lineages. Notably, non-7PET isolates were associated with diarrhea in certain patients, although these strains are generally considered less transmissible and typically linked to milder disease compared to 7PET lineages. MLST profiling revealed that all O1 isolates belong to the pandemic clone ST69, while O139 isolates belong to ST286. However, the MLST profiles of non-O1/O139 *V. cholerae* and *V. paracholerae* were quite diverse. Following this, an ML tree was constructed using 16S, 23S, and 5S rRNA gene sequences to understand the genomic similarity of the study isolates, which differentiated *V. paracholerae* in a distinct clade from *V. cholerae* (Fig. 1). Within the *V. cholerae* clade, non-O1/O139 formed a separate cluster with two O139 in between, and all O1 as a single sub-clade with minor SNP differences within it (Fig. 1). The descriptive analysis data are given in Table S1.

### Phenotypic antimicrobial susceptibility testing of *Vibrio* clinical isolates displayed resistance to multiple antimicrobial agents

**Fig 2:**
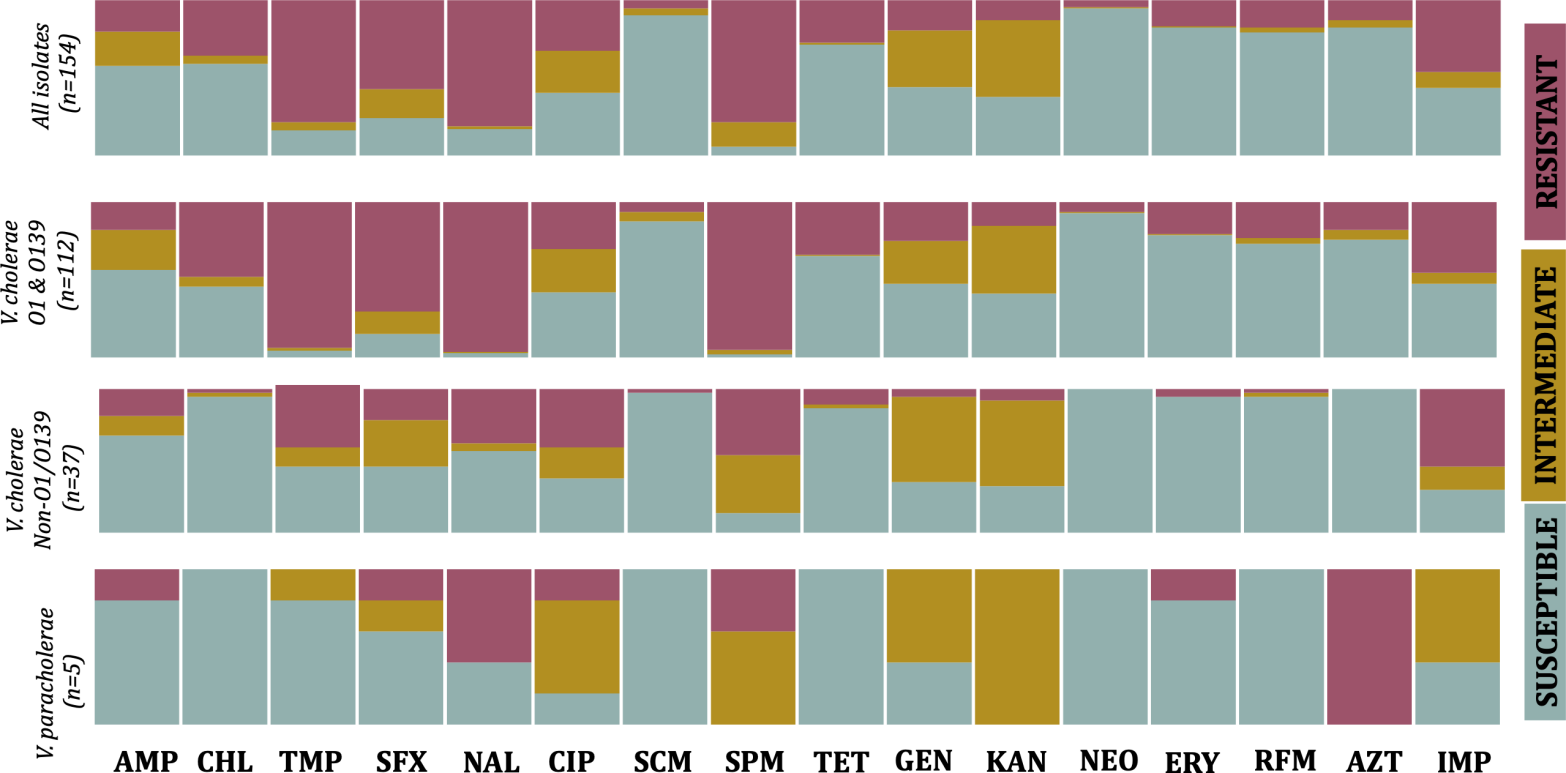
Phenotypic disk-diffusion-based antimicrobial susceptibility profile of the study isolates (*n* = 154) categorized at the level of *Vibrio* species and serogroups. AMP, ampicillin; CHL, chloramphenicol; TMP, trimethoprim; SFX, sulfamethoxazole; NAL, nalidixic acid; CIP, ciprofloxacin; SCM, spectinomycin; SPM, streptomycin; TET, tetracycline; GEN, gentamicin; KAN, kanamycin; NEO, neomycin; ERY, erythromycin; RFM, rifampicin; AZT, aztreonam; and IMP, imipenem.

The cumulative antibiogram of the study isolates tested against commonly used antimicrobials is shown in Fig. 2. Overall, the frequency of resistance, intermediate, and susceptible phenotypes was variable between antimicrobial agents. Resistance was most frequently observed for nalidixic acid, trimethoprim, streptomycin, and sulfamethoxazole, while susceptibility was more frequently observed for spectinomycin and neomycin. Subset analysis revealed a significant difference, with the number of resistance occurring in *V. cholerae* O1 and O139 serogroups generally higher than in non-O1/O139 *V. cholerae* and *V. paracholerae* isolates. In particular, most if not all O1 and O139 *V. cholerae* isolates were resistant to trimethoprim, nalidixic acid, and streptomycin, whereas most non-O1/O139 and *V. paracholerae* isolates were not resistant or had an intermediate resistance. In contrast, *V. paracholerae* isolates were resistant to aztreonam, whereas only a subset of the *V. cholerae* isolates was resistant.

When stratified by serogroup, O1 isolates consistently exhibited higher resistance proportions compared to non-O1/O139 isolates for trimethoprim, nalidixic acid, streptomycin, sulfamethoxazole, and chloramphenicol. Across the 16-year study period (2008–2023), resistance profiles were largely stable within both groups. Among O1 isolates, chloramphenicol resistance demonstrated a declining trend over time, whereas streptomycin resistance showed a modest upward trend. However, for most other antibiotics, including trimethoprim, sulfamethoxazole, nalidixic acid, and ciprofloxacin, no consistent year-on-year increase or decrease was observed. In non-O1/O139 isolates, resistance frequencies were generally lower and did not demonstrate statistically robust temporal trends across the study period.

### Profiling of chromosomal and acquired antimicrobial-resistant determinants among *Vibrio* spp. of diverse origins

Analysis of AMR gene distribution in ML tree highlighted *V. cholerae* O1 as a drug-resistant lineage with multiple drug resistance (MDR) genes, unlike non-O1/O139 *V. cholerae* and *V. paracholerae* (Fig. 1). The chromosomally encoded *catB9* gene was present in all except two O1 isolates, and 3/37 of non-O1/O139 strains and was absent from all O139 and *V. paracholerae* isolates. Furthermore, the SXT-ICE, which harbors a MDR cassette containing the *dfrA*, *floR*, *sul2*, *strA*, and *strB* resistance genes, was present in most of the O1 lineages, even if the cassette does not contain all the determinants, but was absent in *V. cholerae* O139 and *V. paracholerae*.

Although several non-O1/O139 isolates carried individual resistance genes, the fragmented nature of their draft assemblies limited our ability to determine whether these genes were associated with SXT-ICE. Consistent with this, analysis of the six non-O1/O139 genomes showed only limited ICE-SXT similarity (3%–7.5%) to the 97 kb SXT-ICE reference, except isolate S49_R64, which showed 56% coverage. However, BLAST analysis of AMR gene-containing contigs revealed no extended similarity to the SXT-ICE backbone beyond the resistance loci, which were instead flanked by transposase genes, suggesting association with alternative mobile genetic elements.

To further validate ICE status, raw reads from each isolate were mapped to the SXT-ICE reference, and mean coverage was calculated in 1 kb bins across the backbone. Based on coverage continuity and depth, isolates were classified as full ICE (continuous high-depth coverage), partial ICE (discontinuous or region-restricted coverage), or absent ICE (near-zero coverage). Full ICE coverage was predominantly observed among O1 isolates, whereas non-O1/O139 isolates were restricted to partial or absent categories; O139 isolates displayed intermediate patterns. Importantly, no non-O1/O139 isolate demonstrated continuous backbone coverage consistent with a complete ICE. These read-based results confirm that AMR genes in non-O1/O139 isolates are not embedded within an intact SXT-ICE structure (Fig. S1).

A few non-classical AMR determinants were identified; *qnrVC*1 was exclusively present in *V. cholerae* O1 (*n* = 8) and non-O139 strains (*n* = 3), while *bla_CARB-9_* was present in both *V. cholerae* non-O1/O139 (*n* = 3) and *V. paracholerae* (*n* = 4). However, upon further examination within the 7PET O1 lineage, all *qnrVC1*-positive isolates were found exclusively among early El Tor wave strains and were absent in late wave 3 isolates. This indicates that *qnrVC1* distribution within O1 is associated with specific El Tor variant waves rather than being uniformly disseminated across the entire O1 population.

Chromosomally mediated resistance was common in both clinical *Vibrio* species. Fluoroquinolone resistance caused by double mutations in *gyrA* (S83I) and *parC* (S85L) was more prevalent in *V. cholerae* O1 compared to non-O1/O139 strains. However, four of the five *V. paracholerae* strains showed mutations in *gyrA* and *parC*, highlighting the higher prevalence of quinolone resistance in *V. paracholerae* also.

### Linking phenotypic resistance to AMR determinants

**Fig 3:**
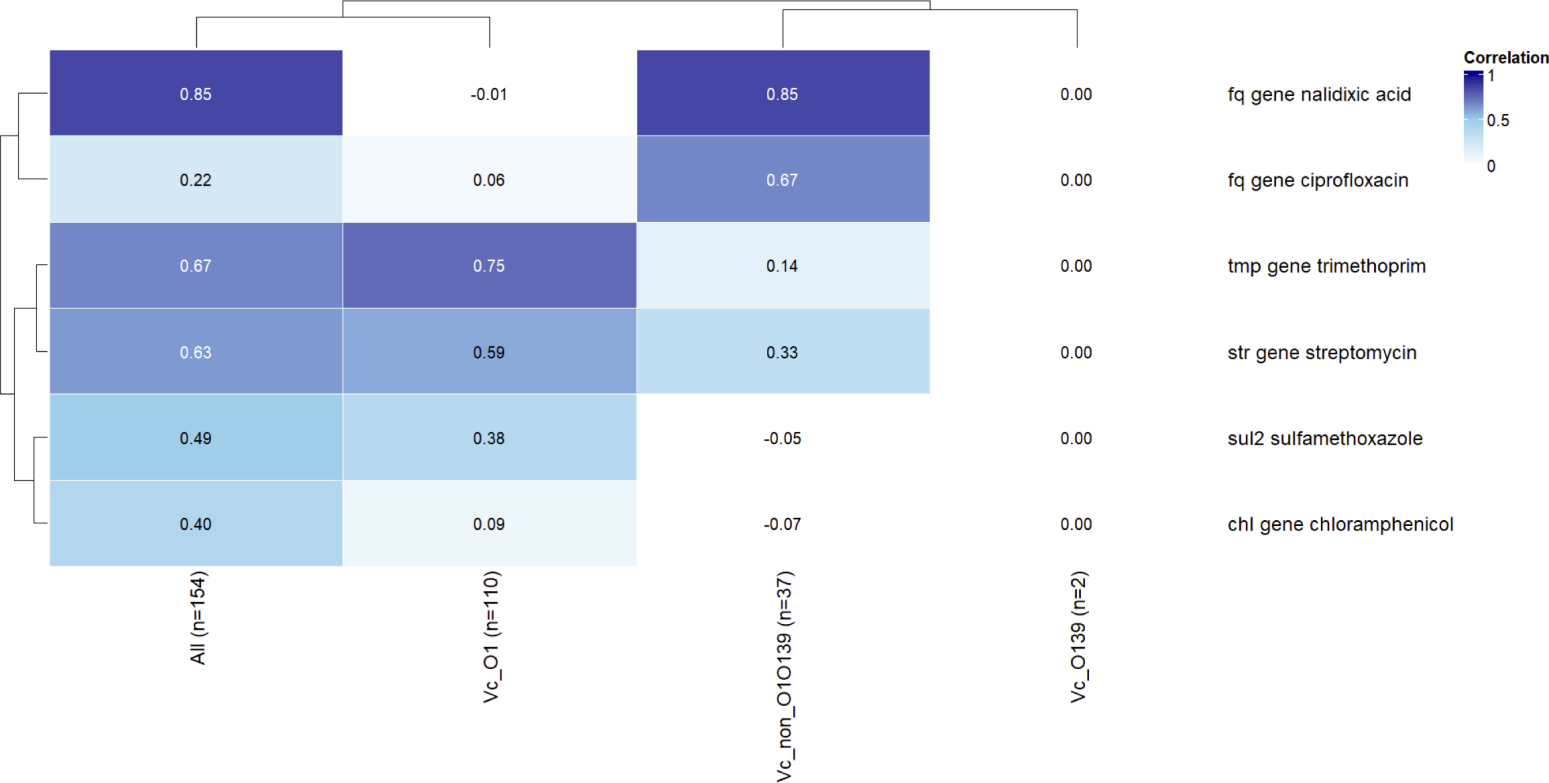
Heatmap showing Pearson correlation coefficients between combined AMR gene indicators and phenotypic resistance outcomes for all isolates of *Vibrio cholerae* and for each serogroup category. Higher values (toward dark blue) indicate stronger agreement between genotype and phenotype.

Genotype–phenotype concordance varied substantially across antibiotic classes, depicted in the heatmap (Fig. 3). The strongest agreement was observed for nalidixic acid, where *gyrA/parC* mutations produced high TP rates (TP = 124) with minimal discordance (FP = 6 and FN = 1). Trimethoprim also showed good concordance using combined *dfrA1/dfrA15* markers (TP = 105 and FP = 4). Streptomycin (*strA/strB*) and sulfamethoxazole (*sul2*) demonstrated moderate agreement. In contrast, chloramphenicol resistance determinants (*catB9/floR*) were frequently detected in susceptible isolates, resulting in numerous false positives (FP = 61), indicating poor predictive utility. Ciprofloxacin similarly showed substantial discordance with *gyrA/parC* mutations (FP = 82), likely reflecting partial reductions in susceptibility that do not exceed ciprofloxacin breakpoints.

### Global contextualization of the phylogenomic analysis highlights a clear clustering of *Vibrio* spp. with high diversity within the species

**Fig 4:**
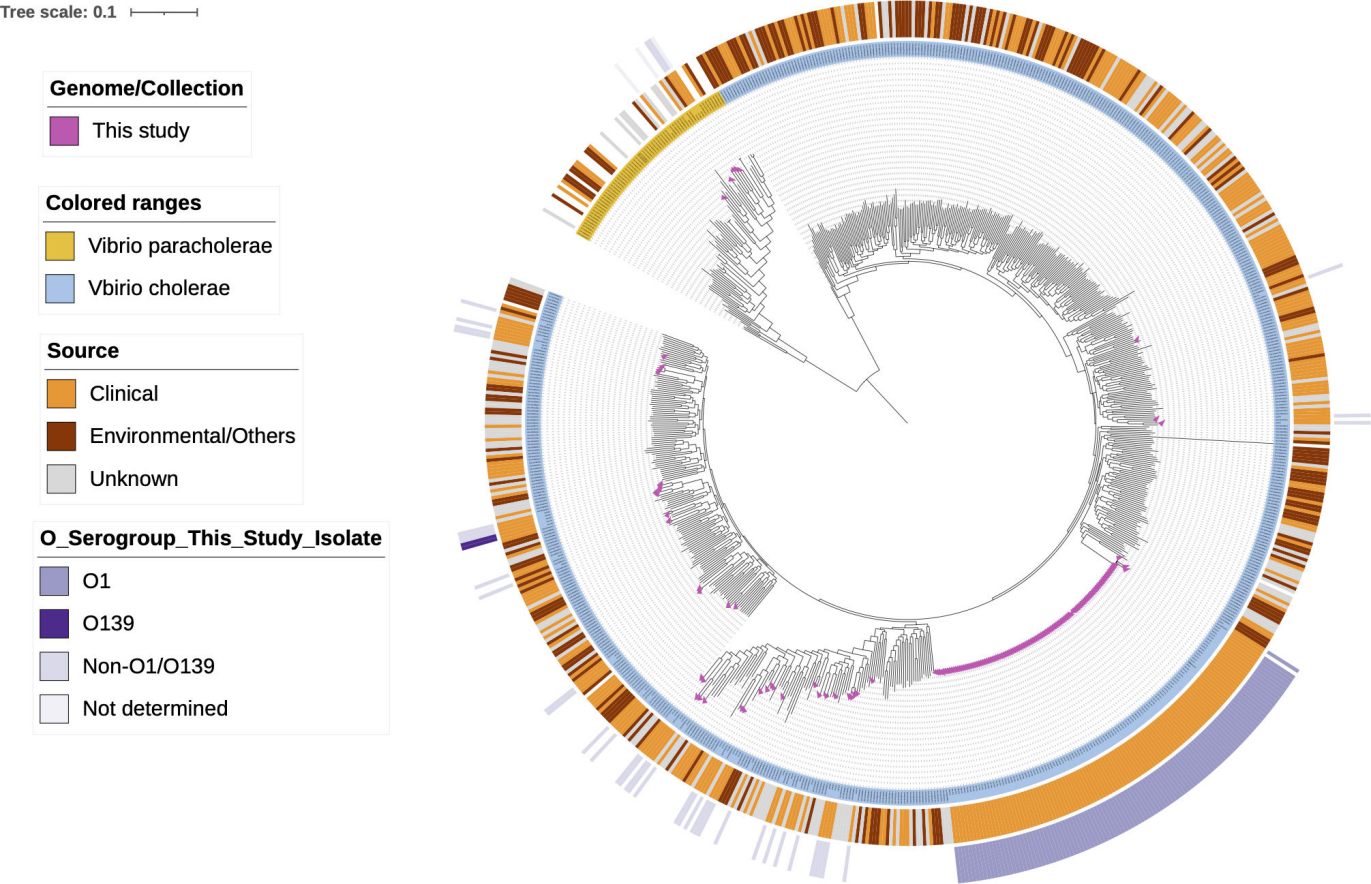
Single nucleotide polymorphism (SNP) based maximum likelihood phylogenetic tree based on 2,678 core genes obtained by panaroo analysis. The color strip indicates the present study isolates, *Vibrio* spp., source, and *Vibrio* serogroups identified based on the O-antigen markers. The scale bar indicates the number of substitutions per site per genome.

To place our study isolates in a global context, we curated a data set of ~6,311 *V*. *cholerae* genomes from the NCBI Pathogen Detection database. Using the dereplicator tool, we further selected 570 representative *V. cholerae* genomes. We also incorporated 31 *V. paracholerae* assemblies from the NCBI database, creating a data set of 601 global genomes, to which we added our 154 isolates. Given the inclusion of two different species, a core-genome-based ML tree was constructed, which revealed two distinct clades; one comprising *V. cholerae* and the other *V. paracholerae* (Fig. 4). Metadata mapping on the ML tree highlighted the distinct clustering of O1 isolates as a single lineage, with two O139 genomes and scattered non-O1/O139 genomes across the tree. All *V. paracholerae* isolates formed a cohesive cluster, indicating their genetic similarity. The core genome phylogeny showed a mingling of isolates from different origins, with small clusters reflecting their respective clinical and environmental sources.

### Pan-genome comparisons identify distinct species-specific regions in *V. paracholerae* and *V. cholerae*

**Fig 5:**
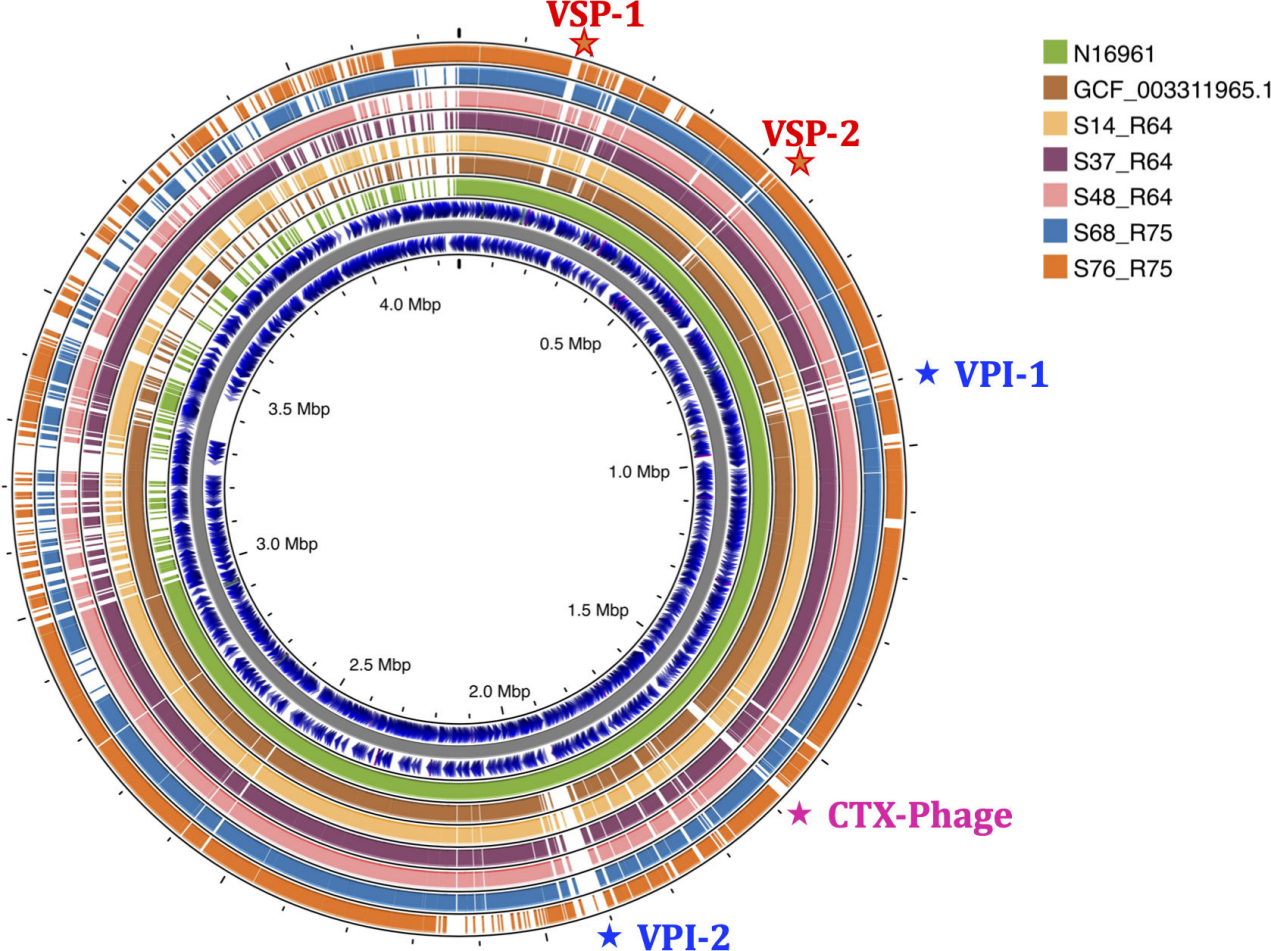
Circular map represents the comparison of *V. paracholerae* (*n* = 5) against the reference genomes of *V. cholerae* N16961 and *V. paracholerae*. The presence or absence of regions indicates the differences between the two species. This figure was generated using the Proksee tool.

To characterize the genetic differences between *V. cholerae* and *V. paracholerae*, we performed a genome-genome comparison between the well-studied O1 reference genome of N16961, the *V. paracholerae* reference (GCA_003311965), and the five isolates of *V. paracholerae* from this study using BLAST-ATLAS tool. The pan-genome gbk obtained from the BLAST-ATLAS was used as a mapping reference, showing the presence or absence of genes/regions across the genome. Figure 5 illustrates the presence of genomic regions in the N16961 genome, but absent in all of the *V. paracholerae* genomes or vice versa. Notably, *V. cholerae* N16961 carried VPI-1, VPI-2, VSP-1, VSP-2, and CTXΦ regions that were absent in *V. paracholerae*. In contrast, regions/genes that are present in all *V. paracholerae* but absent in O1-N16961 genomes were predominantly annotated as hypothetical proteins. Further analysis using Phandango revealed distinct gene presence/absence patterns for *V. cholerae* O1 isolates, especially for O-antigen biosynthesis and 7PET-related islands (Fig. S2). In contrast, the gene flow matrix plotted for all *V. paracholerae* collected globally with the study isolates showed no significant differences (Fig. S3).

### Proteogenomic analysis among *V. cholerae* and *V. paracholerae* revealed differential expression of virulence genes

**Fig 6:**
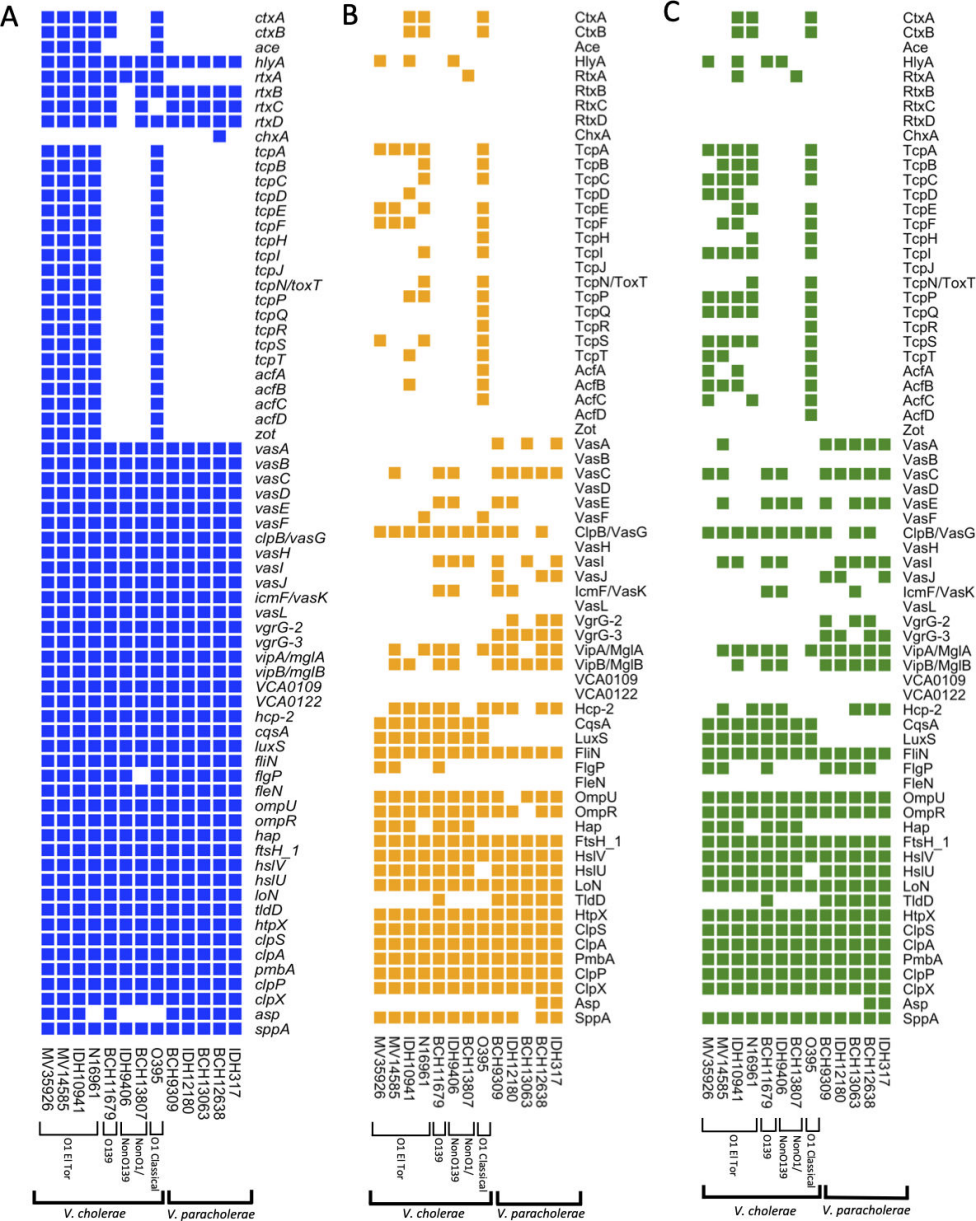
The heatmap illustrates the virulence pattern in *V. cholerae* (*n* = 8) and *V. paracholerae* (*n* = 5) strains based on comparative genomic and proteomic analysis: (**A**) the presence (blue) and absence (white) of virulent genes, (**B**) expression levels (orange) of virulent genes in LB media, and (**C**) expression levels (green) of virulent genes in AKI media (see also Table S2).

Despite lacking the key CtxAB, no new toxin gene seems to be present in *V. paracholerae* compared to *V. cholerae* genomes. To explore potential virulence factors involved in cholera-like disease, we performed a proteogenomic analysis of virulence-related genes in the five *V. paracholerae* and eight *V. cholerae* isolates. The qualitative expression of virulence genes was examined in LB and AKI media for comparative proteomic analysis (Table S2). Virulence database, NCBI BLAST, and proteomic experiments confirmed that genes encoding for CtxA and CtxB, the two major cholera enterotoxin subunits, were present in all four O1 El Tor, O1 classical, and O139 strains. However, all *V. paracholerae* and non-O1/O139 *V. cholerae* strains lacked these genes (Fig. 6). Accessory cholera enterotoxin *ace* was present in El Tor strains and in the O1 classical strain VCO395, although expression was not detected, due to its differences in induction conditions (29). The hemolysin gene (*hylA*) was present in all strains. The El Tor and non-O1/O139 strains expressed *hylA* in both LB and AKI, but for O139, the expression was detected only in AKI media. The RTX toxin system was present in all El Tor strains, O139, and one non-O1/O139 strain (BCH13807). For *V. paracholerae* isolates, *rtxA* showed a partial BLAST hit, but *rtxBCD* were conserved. Similarly, cholix toxin *chxA* was present in only one strain of *V. paracholerae* (BCH12638). Genes encoding for toxin co-regulated pilus (*tcp*) biogenesis and the AcfABCD accessory colonization factors were exclusively present in El Tor and classical strains. Likewise, genes encoding for Type VI secretion system (T6SS), phage-tail like components TssA (*vasJ*), TssB (*vipA/mglA*), TssC (*vipB/mglB*), hemolysin co-regulated protein Hcp (*hcp-2*), phage tail spike like protein VgrG (*vgrG*), and ATP degrading protein ClpV (*clpB*/*vasG*) were found in all *V. cholerae* and *V. paracholerae* isolates. The expression of T6SS was higher in AKI medium with the exception of *icmF/vasK*, *vgrG-3*, and *hcp-2* genes. Genes of different proteases were present in all isolates, except alkaline serine protease (*asp*) that was absent in non-O1/O139 strains and classical strains (Fig. 6A; Table S2).

### Characterization of the chromosomal dimer resolution locus (*dif1*) in *V. paracholerae* and its incompatibility with CTXΦ integration

The cholera toxin genes are part of the CTXΦ genome, which *V. cholerae* has acquired and integrated into its genome at the dimer resolution locus (*dif*) via the bacterial Xer recombinase machinery. In addition to CTXΦ, *V. cholerae* harbors various Integrative Mobile Elements exploiting Xer (IMEXs), such as the toxin-linked cryptic element (TLC), VJGΦ, and RS1, which contribute to pathogenicity by aiding toxin acquisition and dissemination.

We analyzed the functional integration sites and copy numbers of the TLC, CTX prophage, and RS1 elements in *V. cholerae* and *V. paracholerae* genomes. Using the *zot* gene as a proxy for CTX copies, *rstC* for RS1 copies, and *xafT/cri* for TLC elements, we determined the presence of IMEXs in the *dif* locus. We mapped prophage integration sites by identifying conserved *dif1* sequences at the terminus site of chromosome 1. Table 1 summarizes the *dif1* sequences identified in *V. cholerae* and *V. paracholerae* strains in relation to presence or absence of CTXprophage. The *V. cholerae* O1 El Tor (N16961) and classical (O395) strains, both carrying the CTX-prophage, possess *dif1* sequences compatible with the phage integration. In contrast, non-toxigenic *V. cholerae* strains such as H25 and WO5, and all *V. paracholerae* isolates analyzed in this study share an identical *dif1* sequence (AGTGCGTATTA GGTATA TTATGTTAAAT). Notably, the cross-over region of *dif* sequences in *V. paracholerae* contains a guanine (G) immediately adjacent to the XerC binding site, which prevents XerC-mediated strand exchange and hinders CTXΦ integration. This sequence, previously described as *difG* sequence (28), is conserved among non-toxigenic *V. cholerae* strains and all *V. paracholerae* genomes examined. The conservation of *difG* sequence across all *V. paracholerae* genomes explains the universal absence of CTX prophage and associated virulence genes (e.g., *ctxAB*) in this species. This incompatibility explains why *V. paracholerae* lacks cholera toxin genes in its genome. Furthermore, *V. paracholerae* isolates were found to lack the VPI region, which encodes the toxin co-regulated pilus (TCP), the receptor required for CTXΦ entry. These findings further support the genomic incompatibility of *V. paracholerae* with cholera toxin acquisition.

**TABLE 1:** The *dif* sequences in the large chromosome of *Vibrio cholerae* and *Vibrio paracholerae*

Bacterial species	*dif* sequence	CTX-prophage	Reference/source
*Vibrio cholerae* N16961	AGTGCGTATTA TGTATG TTATGTTAAAT	+ve	(28)
*Vibrio cholerae* O395	AATGCGTATTA CGTGCG TTATGTTAAAT	+ve	(28)
*Vibrio cholerae* H25	AGTGCGTATTA **G**GTAT**A** TTATGTTAAAT	−ve	(28)
*Vibrio cholerae* WO5	AGTGCGTATTA **G**GTAT**A** TTATGTTAAAT	−ve	(28)
*Vibrio paracholerae* IDH12180	AGTGCGTATTA **G**GTAT**A** TTATGTTAAAT	−ve	This study
*Vibrio paracholerae* BCH13063	AGTGCGTATTA **G**GTAT**A** TTATGTTAAAT	−ve	This study
*Vibrio paracholerae* BCH12638	AGTGCGTATTA **G**GTAT**A** TTATGTTAAAT	−ve	This study
*Vibrio paracholerae* IDH317	AGTGCGTATTA **G**GTAT**A** TTATGTTAAAT	−ve	This study
*Vibrio paracholerae strain* 2016V-1111	AGTGCGTATTA **G**GTAT**A** TTATGTTAAAT	−ve	GCA_003311965.1
*Vibrio paracholerae* *Strain* VCC19	AGTGCGTATTA **G**GTAT**A** TTATGTTAAAT	−ve	GCA_000438805.2
*Vibrio paracholerae* strain *PGB-VPC004*	AGTGCGTATTA **G**GTAT**A** TTATGTTAAAT	−ve	GCA_024106035.1
*Vibrio paracholerae. Strain PGB-VPC014*	AGTGCGTATTA **G**GTAT**A** TTATGTTAAAT	−ve	GCA_024105875.1

## DISCUSSION

This study provides critical insights into the AMR patterns, genomic diversity, and a comparative proteome profiling of *V. cholerae* and *V. paracholerae* collected over a 16-year period. Phenotypic analysis revealed significant AMR resistance for streptomycin, nalidixic acid, and trimethoprim, especially in *V. cholerae* O1 and O139 serogroups as compared to non-O1/O139 and *V. paracholerae* isolates. In contrast, all *V. paracholerae* showed aztreonam resistance. The resistance patterns are likely driven by SXT-ICE-borne resistance genes and chromosomal mutations in *gyrA* and *parC* conferring quinolone resistance.

Genome-based analysis revealed genetic diversity among *Vibrio* isolates, identifying *V. paracholerae* alongside predominant *V. cholerae* strains across serogroups O1, O139, and non-O1/O139. The identification of different serogroups within *V. cholerae* isolates and their corresponding wave variants offers insights into the epidemiology and temporal dynamics of cholera in India. The AMR profiling revealed that *V. cholerae* O1 is a prominent drug-resistant lineage, carrying the chromosomally encoded *catB9* gene and the SXT-ICE-mediated resistance cassette, features that are absent in *V. cholerae* O139 and *V. paracholerae*. The detection of specific AMR genes like *qnrVC*1 and *bla*_CARB-9_ in *V. cholerae* and *V. paracholerae*, respectively, underscores the diverse resistance mechanisms and the need for continued genomic surveillance.

For the genotype-phenotype concordance analysis, we found several resistance genes failed to predict phenotype, because the presence of AMR genes does not consistently translate into functional resistance. Certain genes (e.g., *catB9* and *floR*) may be transcriptionally silent, poorly expressed, or encode non-functional variants, leading to phenotypically susceptible isolates despite gene carriage. Fluoroquinolone mutations may confer intermediate or borderline MIC increases, insufficient to meet clinical ciprofloxacin resistance thresholds, contributing to false positives. For sulfamethoxazole and streptomycin, additional mechanisms—such as alternative gene variants, target-site mutations, or compensatory pathways—likely account for resistance in gene-negative isolates. Overall, these findings highlight the need for gene-specific interpretation when applying WGS-based AMR prediction in *Vibrio* spp.

Phylogenomic analysis contextualized the global diversity of *Vibrio* spp., revealing distinct clades for *V. cholerae* and *V. paracholerae*. The clustering of *V. cholerae* isolates by serogroup, with *V. paracholerae* forming a separate, cohesive cluster, highlights the species-specific genetic variations. Pan-genome comparisons highlighted unique genomic regions in *V. cholerae*—including VPI-1, VPI-2, VSP-1, VSP-2, and CTXΦ—absent in *V. paracholerae*, with the predominance of hypothetical proteins in *V. paracholerae*. These differences, reflected in the gene presence or absence profiles, provide insights into the evolutionary divergence between these species.

Comparative proteomics supported genomic findings, revealing differential virulence gene expression among *Vibrio* isolates, with overall higher expression in AKI media. While classical cholera toxin genes were absent in *V. paracholerae*, hemolysin was present in both *Vibrio* species with variable expression, and genes encoding the type VI secretion system were conserved across all isolates and expressed variably, suggesting alternative virulence mechanisms. Interestingly, cholix toxin was found in only one *V. paracholerae* strain, indicating potential strain-specific pathogenicity. Pathogenic factors involved in quorum sensing, adherence, motility, and protease activity were detected in both *V. cholerae* and *V. paracholerae*, exhibiting variable expression in both LB and AKI media. These findings suggest that *V. paracholerae* may rely on distinct virulence factors, differing from *V. cholerae*’s classical cholera toxin-mediated pathogenesis.

Despite the modest annual numbers, the data set represents a systematically collected, year-wise sample from an endemic region over 16 years (2008–2023), capturing key temporal shifts in circulating lineages and resistance patterns. Overall, the data suggest persistence of established resistance phenotypes within dominant lineages rather than progressive escalation of antimicrobial resistance over time.

Whole genome analysis revealed that *V. paracholerae* lacks TLC, CTX, and RS1 prophages, as well as conserved integration sites found in toxigenic *V. cholerae*, suggesting it is unlikely to acquire cholera toxin. The genetic differences between *V. cholerae* and *V. paracholerae* highlighted in the present study, along with those reported by Islam et al. (12), can serve as markers for developing rapid diagnostic tests to differentiate between the two species. Thus, this study emphasizes the importance of disease surveillance in cholera-endemic countries like India and highlights the need for accurate identification and discrimination of closely related species in clinical samples.

## Data Availability

All genomic data can be accessed through Indian Biological Data Center (IBDC), accession number INRP000309 and the National Center for Biotechnology Information (NCBI) database under BioProject accession number PRJNA1193462, and the proteomic data are available in ProteomeXchange; PRIDE accession number PXD057625.

## SUPPLEMENTAL MATERIAL

Figure S1 spectrum.02162-25-s0001.pdf Heatmap showing mean read depth across the reference SXT-ICE backbone, calculated in non-overlapping 1 kb bins.

Figure S2 spectrum.02162-25-s0002.pdf Pan-genome profile of *Vibrio* spp. constructed using Phandango indicates the gene flux as presence or absence matrix.

Figure S3 spectrum.02162-25-s0003.pdf Pan-genome profile of *V. paracholerae* of this study isolates and the global collection constructed using Phandango indicates the gene flux as presence or absence matrix.

Supplemental legends spectrum.02162-25-s0004.pdf Legends for Figures S1 and S3, and Tables S1 and S2.

Table S1 spectrum.02162-25-s0005.xlsx Details of *Vibrio cholerae* and *Vibrio paracholerae* strains isolated, characterized, sequenced, and analyzed in the current study.

Table S2 spectrum.02162-25-s0006.xlsx Details of the virulence gene profiles and their corresponding protein expression levels in Luria Bertani (LB) and Alkaline Peptone Infusion (AKI) media.

ASM does not own the copyrights to Supplemental Material that may be linked to, or accessed through, an article. The authors have granted ASM a non-exclusive, world-wide license to publish the Supplemental Material files. Please contact the corresponding author directly for reuse.
