# Supplementary figures and images for: A decade of antimicrobial resistance in Vibrio spp.: genomic and functional insights
Source: Microbiol Spectr. 2026 Apr 2;14(5):e02162-25. doi: 10.1128/spectrum.02162-25 (PMC13142030; doi:10.1128/spectrum.02162-25)

Serogroup  
O1 O139 Non-O1/O139

SXT-ICE Read Depth Heatmap with ICE Groups and Serogroup Annotation

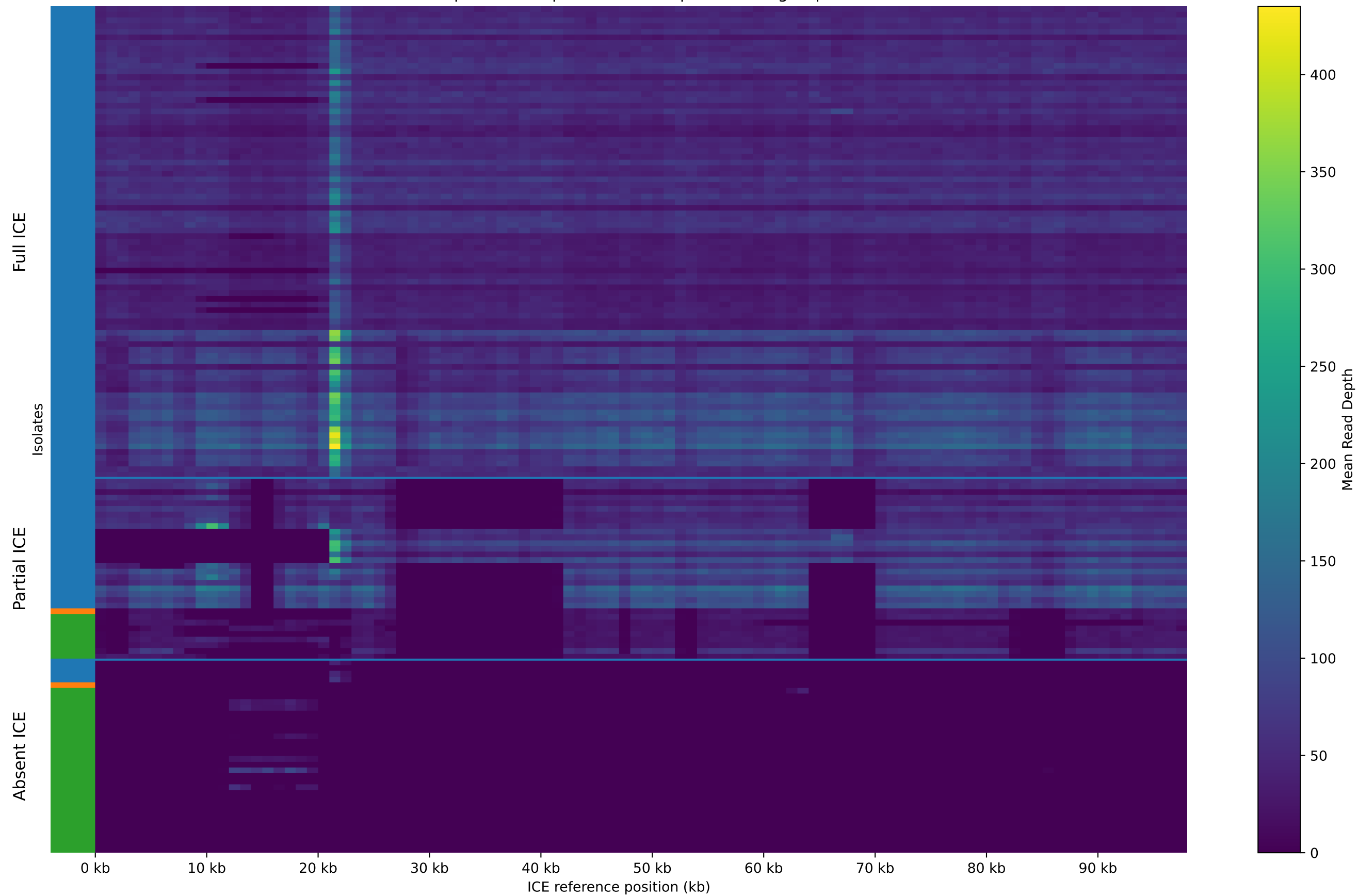

Supplement: Figure S1 — Heatmap showing mean read depth across the reference SXT-ICE backbone, calculated in non-overlapping 1 kb bins. [file spectrum.02162-25-s0001.pdf]

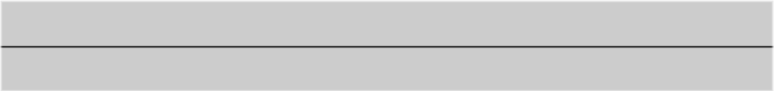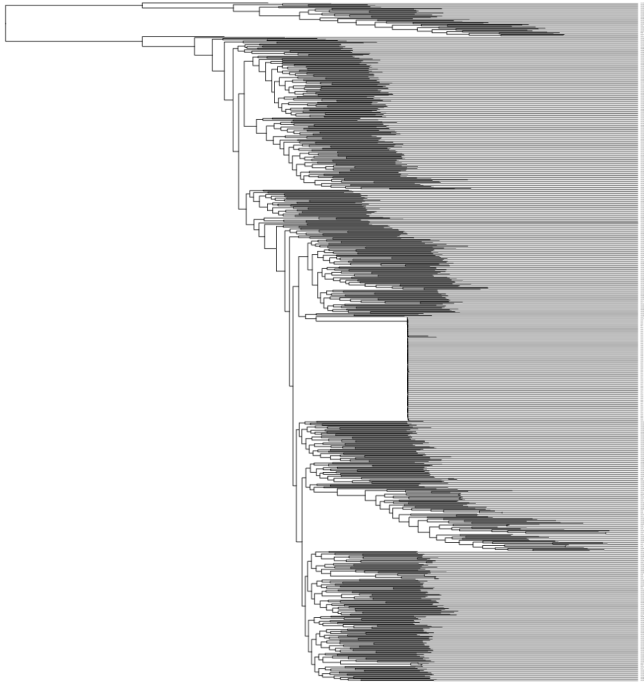

Species  
Collection  
Year  
Source  
Location

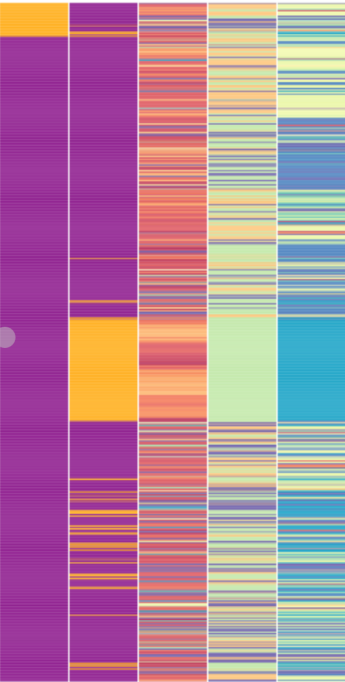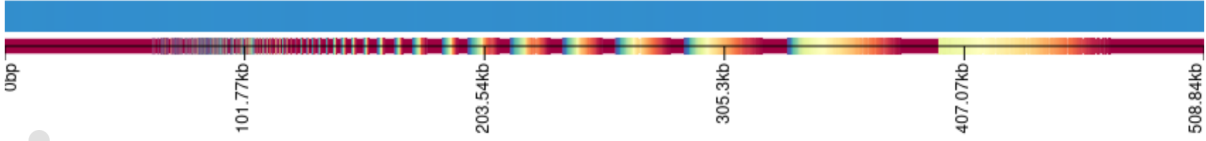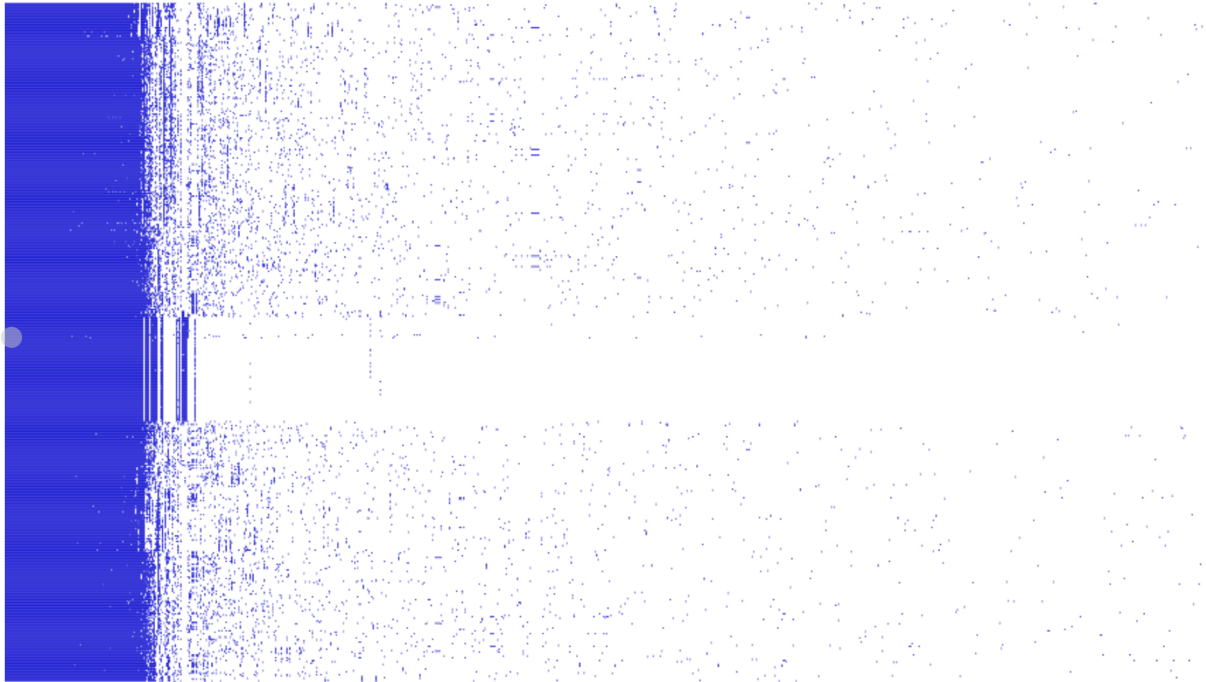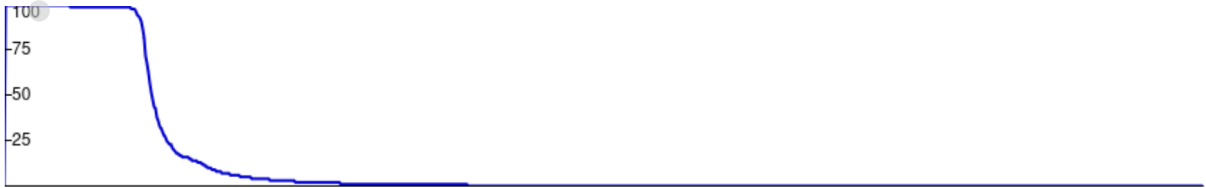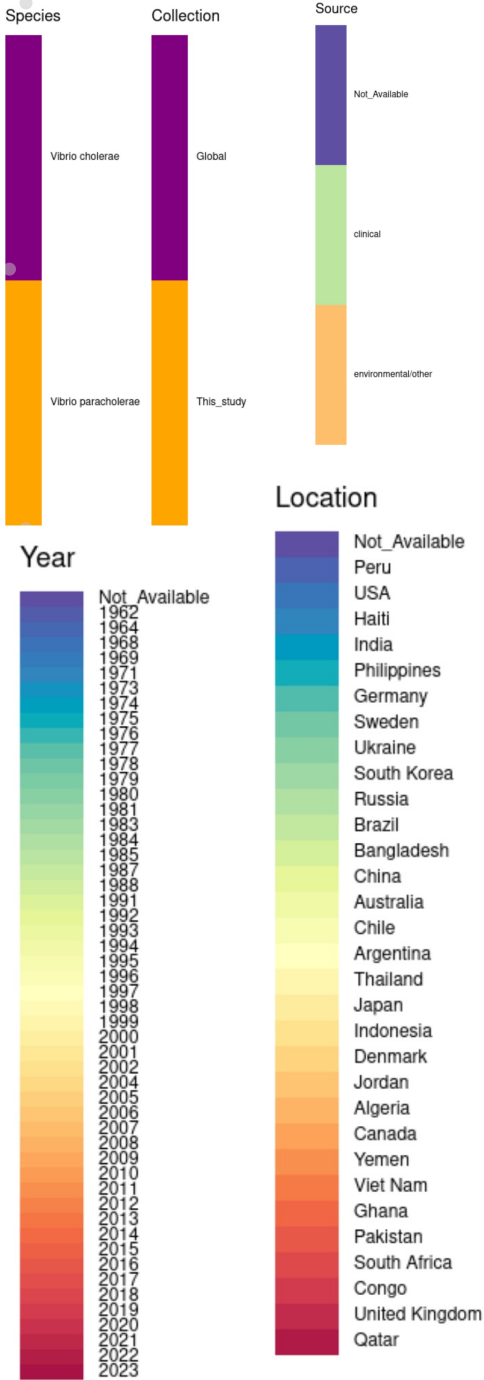

Supplement: Figure S2 — Pan-genome profile of Vibrio spp. constructed using Phandango indicates the gene flux as presence or absence matrix. [file spectrum.02162-25-s0002.pdf]

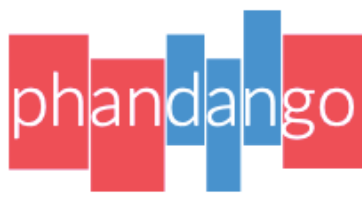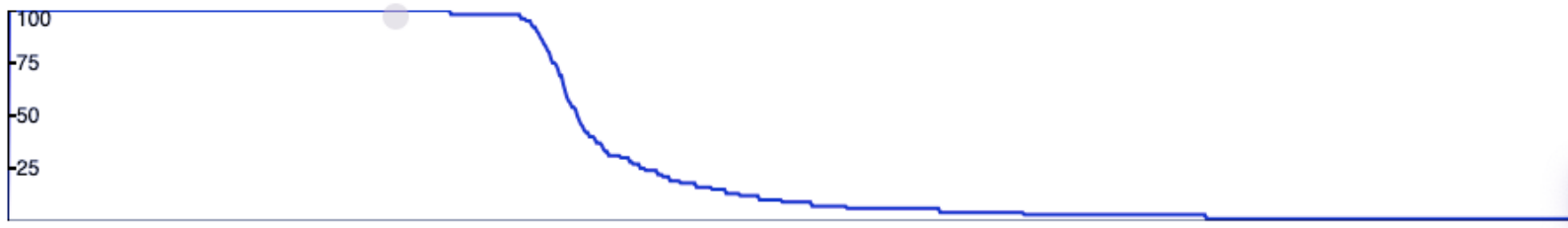

Supplement: Figure S3 — Pan-genome profile of V. paracholerae of this study isolates and the global collection constructed using Phandango indicates the gene flux as presence or absence matrix. [file spectrum.02162-25-s0003.pdf]
