# Supplementary material for: A decade of antimicrobial resistance in Vibrio spp.: genomic and functional insights
Source: Microbiol Spectr. 2026 Apr 2;14(5):e02162-25. doi: 10.1128/spectrum.02162-25 (PMC13142030; doi:10.1128/spectrum.02162-25)
Supplement: Supplemental legends — Legends for Figures S1 and S3, and Tables S1 and S2. [file spectrum.02162-25-s0004.pdf]

A Decade of Antimicrobial Resistance in *Vibrio* spp.: Genomic and Functional Insights

Agila Kumari Pragasam[1#], Chandana Basak[1#], Mansi Rajwanshi[1], Lekshmi Narendrakumar[1], Goutam Chowdhury[2], Prabhakar Babele[1], Meenal Chawla[1], Orlan do Moranchel[3], Upma Singh[1], Deboleena Roy[2], Pradipta Jana[1], Tanshi Mehrotra[1], Debaleena Das[1], Daizee Talukdar[1], Deepjyoti Paul[1], Rupak K Bhadra[4], Thandavarayan Ramamurthy[2], Amit Ghosh[2], Shanta Dutta[2], Christophe Possoz[3], Francois-Xavier Barre[3], Asish K Mukhopadhyay[2], Bhabatosh Das[1@]

Supplementary information

Description of Supplementary Tables:

Supplementary Table S1. Details of Vibrio cholerae and Vibrio paracholerae strains isolated, characterized, sequenced, and analyzed in the current study, including strain identifiers, source of isolation, and key genomic and phenotypic characteristics.

Supplementary Table S2. Excel file provides details of the virulence gene profiles and their corresponding protein expression levels in Luria Bertani (LB) and AKI media, as related to Figure 5. Gene presence and protein expression are denoted by "1", while gene absence and lack of protein expression are denoted by "0".

Legend to Supplementary Figures:

Supplementary Figure S1. Heatmap showing mean read depth across the reference SXT-ICE backbone, calculated in non-overlapping 1 kb bins. Raw sequencing reads from each *V. cholerae* study isolate were mapped to the SXT-ICE reference sequence, and per-base coverage was summarized to generate the coverage matrix. Isolates are grouped according to ICE status based on coverage continuity and depth: Full ICE (continuous high-depth coverage across the backbone), Partial ICE (discontinuous or region-restricted coverage), and Absent ICE (near-zero coverage across the region). A side annotation bar indicates serogroup classification (O1, O139, Non-O1/O139). *V. paracholerae* isolates were excluded from this analysis. Solid horizontal lines demarcate ICE status groups. The color scale represents mean read depth.

Supplementary Figure S2. Pan-genome profile of *Vibrio* spp. constructed using Phandango indicates the gene flux as presence or absence matrix. Color strip indicates the respective metadata associated with the genomes as *Vibrio* spp. year, location and source as given in the legend. Dark blue indicates the presence of genes.

Supplementary Figure S3. Pan-genome profile of *V. paracholerae* of this study isolates and the global collection constructed using Phandango indicates the gene flux as presence or absence matrix. Color strip indicates the respective metadata associated with the genomes as *Vibrio* spp, year, location and source as given in the legend. Dark blue indicates the presence of genes.
